# Identifying the Perceptual Drivers to Attract Attention and Enhance the Choice to Purchase Pearl Millet

**DOI:** 10.3390/foods15152706

**Published:** 2026-07-31

**Authors:** Chantelle Fourie, Elizabeth Kempen

**Affiliations:** Department of Life and Consumer Sciences, University of South Africa, Florida Science Campus, Roodepoort 1710, South Africa; chantellefourie95@gmail.com

**Keywords:** food insecurity, indigenous foods, sustainable agriculture, consumer consumption, locally produced foods

## Abstract

Indigenous grains, such as pearl millet, can contribute to alleviating food insecurity in South Africa and addressing the health and well-being of consumers. Stigmatisation and a lack of consumer knowledge about indigenous grains have been found to stifle the consumption of these grains. Therefore, this study aimed to explore South African consumers’ perceived understanding and experience of pearl millet and the external product attributes that attract their attention and steer them towards purchasing and consuming this indigenous grain. This interpretivist phenomenological exploratory study used a qualitative methodology. Small synchronous online focus groups were used to gather the data. The thematic analyses revealed that consumers are generally perceived as uncertain about and lacking experience in the use and consumption of pearl millet, which results from factors such as product unfamiliarity, a lack of knowledge, the product being overlooked in stores, and various assumptions about the grain. Consumers’ perceived willingness to purchase pearl millet may be influenced by several product attraction indicators, including price, packaging, whether it is produced locally, retailer image, and quality-enhancement attributes, which marketers and product developers in South Africa should use to grow consumer interest. This study contributes towards changing the consumer perceived approach to pearl millet by identifying the factors that hamper and limit the choice of pearl millet consumption. South Africa can ill afford to neglect marketing and improving consumer awareness of pearl millet if the health and well-being of South African consumers are to be improved.

## 1. Introduction

### 1.1. Contextual Background About Pearl Millet

The South African population continuously faces food security, and health and well-being challenges. Sustainable Development Goal 2 (SDG2), supported by Sustainable Development Goal 3 (SDG3), is aimed at improving nutrition to end hunger and advancing food security and sustainable agricultural practices to support healthy lives by 2030 [1]. The cultivation of indigenous foods as local resources in South Africa and their consumption could advance both SDGs. Indigenous foods are a part of indigenous knowledge systems and practices that have been developed and accumulated over generations and are an integral part of the unique food culture of local communities in Africa [2,3]. These foods are known to be resilient and better adapted to marginal agricultural contexts; require low production inputs; and can potentially improve nutrition, increase dietary diversity [4], and address the triple burden of malnutrition [5]. Unfortunately, many indigenous and indigenised foods, such as pearl millet (Pennisetum glaucum), are underutilised and unable to contribute significantly to these two critical SDGs, even though they are still advocated as vital cereal crops to address food security [6,7]. Although these foods were historically used and grown intensively [8], their use has become less important among modern-day consumers [9]. The absence of these foods from commodity markets [4] has contributed to their neglect and dismissal, resulting in them becoming less known to the modern-day consumer, who has very little to no experience in using them. This lack of consumer knowledge, product experience, and recognition of the potential health and nutritional benefits of indigenous foods inadvertently manifests in consumer non-consumption [10,11], threatening the patronage of interest in, choice to purchase, and use of pearl millet, which negatively influences the advancement of SDG2 and SDG3 in South Africa.

#### 1.1.1. Origins of Pearl Millet and Consumption Practices

**Figure 1:**
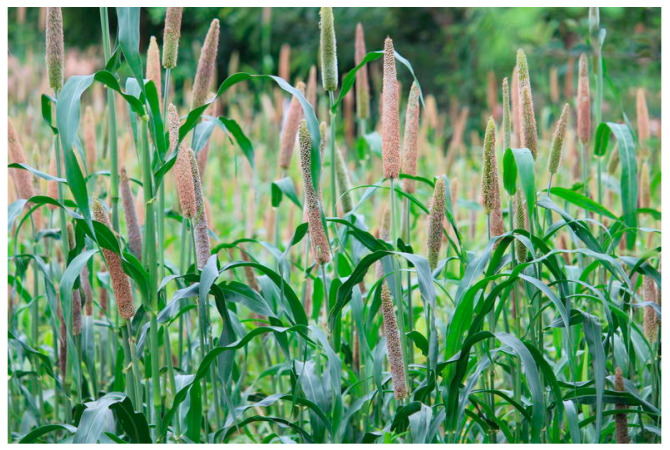
Pearl millet (sourced from https://southafrica.co.za/what-is-pearl-millet.html, 9 June 2026).

Pearl millet (Figure 1), which originates from wild West African grass [12,13,14] and is commonly known as a ‘small grain’ in South Africa [15], is the sixth major cereal crop, alongside maize, rice, wheat, barley, and sorghum, and the third major indigenous grain crop cultivated in Africa [16]. Resulting from its development in semi-arid tropical regions [17], it remains undervalued and unrecognised as a food source in harsh climates and for its drought tolerability, pest resistance, short growing season [15,18], and environmental resistance that add to environmentally friendly farming methods which are beneficial to consumers [19]. As part of ethnically native varieties of traditional food crops consumed in specific regions and by specific groups of individuals, it is a staple food of many communities [19], as with *Omalodu* (a traditional beer from Namibia) [20] and *Fura* (which is made from fermented and non-fermented pearl millet flour and consumed in Nigeria) [21], which are region-specific products that are unfamiliar to many consumers. In South African semi-arid conditions, pearl millet is produced at a sustenance level by smallholder subsistence farmers [15] in poverty-stricken rural regions [5,22], where it is an essential nutritional food source [23]. Pearl millet is consumed mainly as a thick porridge or beverage [5,15], or in an assortment of traditional local confectionary items and flatbreads [5,21]. It has been elevated to a sought-after nutritional source among health-conscious elite consumers [4], to whom it is available as breakfast bars, hulled or dehusked non-GMO (genetically modified organism) millet or millet flour, and in other products in South African retail outlets at a premium price [24] to what [25] found happened with a sorghum product developed in South Africa for general consumer consumption. In recent years, pearl millet-based food bars have gained niche market momentum as a convenience food, considered nutritionally beneficial and appealing when in the form of protein bars [26], and continue to contribute to functional food development, serving modern-day health and well-being [27]. That does not mean that pearl millet is consumed specifically for the nutritional value it adds to such bars, but possibly more for the lifestyle it indirectly supports. Ignorant plant-based health-conscious consumers may in part be the reason for the increased demand for such lifestyle products and not their indigenous nature and inherent nutritional value stifling the consumption of pearl millet [28]. Pearl millet is not marketed as a grain with beneficial properties for the poor, but rather as an ingredient included in speciality food products, branding pearl millet as an expensive health food that is out of the reach of many South African consumers. On the other hand, even if pearl millet were introduced in general food retail outlets, consumers may know too little about it and other indigenous food products to recognise the perceived benefit of purchasing such products out of choice.

#### 1.1.2. Nutritional Importance of Pearl Millet

Pearl millet remains a rich source of energy, vitamin B, and micronutrients [21,29], rendering it superior to maize [30] because it is higher in fibre and more fibre-digestible than maize [31]. It qualifies as a source of protein [32], with a higher lysine content (an essential amino acid) than finger millet or sorghum [33], and has been dubbed ‘today’s nutri-cereal’ [34]. Pearl millet is acknowledged as a functional food and a good source of antioxidants, such as glycated flavonoids and phenolic acids, rendering it a nutraceutical [35] and one of the staple crops for biofortification through which micronutrient deficiencies can be addressed [36]. As a gluten-free grain, it can be consumed as a porridge or used as a flour in flatbread [37], and it is becoming increasingly popular in the baking and snack industries to address the increased demand for gluten-free products [38] and recognised as a future source of edible oil [39] because cereal lipids contain valuable essential fatty acids [35]. Unfortunately, both the primary and secondary processing of millet to enhance digestibility and nutrient bioavailability for consumption purposes reduce the nutrient content of the grain [34]. The concern is that the higher nutritional value of pearl millet in comparison with other cereals (maize, wheat, sorghum, and rice) [40], its potential to contribute to food and nutrition security [41], and its qualities that help address modern-day noncommunicable diseases [42] will remain underutilised if it is not elevated to an acknowledged indigenous grain and consumed in the way exotic grains such as maize are utilised and chosen to be consumed.

### 1.2. Consumer Consumption of Pearl Millet as an Indigenous Grain

A consumer perspective on the underutilisation of indigenous foods is attributed to a general lack of consumption and use knowledge; a lack of market access to such food products, negative perception status [43]; and a nostalgic and ethnocentric approach to indigenous food products, rendering them foreign [10] and causing consumer hesitation to pay for, choose, and consume these products [44]. Cultural diversity in South Africa has further advanced the underutilisation of indigenous foods because not all cultural groups are equally familiar with every type of indigenous food found in the country, resulting in these foods being referred to as ‘poor man’s food’, ‘poverty food’, and ‘backwards-knowledge foods’ [3,45]. The historical past of indigenous grains, as part of rural subsistence lifestyles, where low incomes necessitated sourcing cheap, readily available food crops, resulted in these foods being branded as old-fashioned and still recognised as more prevalent in poor rural communities [10,46]. This does not make these foods less valuable. Urbanisation and exotic-food exposure have turned the modern consumer away from the consumption of indigenous foods. Little indigenous knowledge has been transferred to young urban consumers, raising concerns about the lack of knowledge about these food products and their use by this generation [9,45], who either does not use these products or infrequently uses indigenous foods to the point that they contribute minimally to their nutritional intake [47]. Omotayo and Aremu [48] found that even if consumers in rural households had considerable knowledge of indigenous foods, it did not necessarily lead to the cultivation or consumption of these foods.

Consumer product-related expectations [49], familiarity with indigenous foods [44], existing demographic and socio-economic situations [50], and sensory acceptability when combined with other food products [51] have been related to the willingness to consume indigenous foods. Balogh [44] found that consumers’ willingness to consume these products was influenced by the considerable perceived price premium of indigenous foods compared with other products, with certain retail outlets demanding a higher price and certification protecting the integrity of such foods. Importantly, external product attributes may influence the perceived acceptance of indigenous food products greatly, as price, packaging, and quality of a product are known to contribute to its perceived value and thus stimulate the intention to purchase [52]. Similarly, store image and country of origin resonating with the positive emotional purchasing experience of consumers may have a significant influence on the associated offerings and purchase intention [53]. A more general introduction of domestically and commercially developed pearl millet products could, therefore, stimulate indigenous food product consumption [39] and choice, thus improving the market potential of pearl millet, which is desperately needed to address food insecurity [54]. However, that would require key marketing strategies to introduce indigenous grains as perceived accessible indigenous food products to the general South African consumer.

Studies by [21,22,33,55,56] were concerned with the nutritional value and health benefits related to the consumption of pearl millet, with [57] recently determining the acceptability of pearl millet as a substitute for whole-wheat bread in a Western culture, conscious of unfamiliar flavours that pearl millet may add to food products. Meanwhile, refs. [4,9,17] have published studies supporting the significance of pearl millet as an underutilised indigenous food source, emphasising the contribution this cereal could make to addressing SDG2 and SDG3. Although researchers constantly emphasise the contribution that pearl millet could make to alleviating food insecurity, research has not focused on whether consumers would choose to purchase and consume pearl millet, what they understand about pearl millet, what experience they have of pearl millet, or what would attract them to the grain. Qualitative research is further lacking in the research on pearl millet regarding how consumers’ lived experiences could be captured. Specifically, a qualitative research methodology using focus groups has not been used to explore South African consumers’ understanding and experience of pearl millet as contributing choice factors in the purchase of the grain. Hence, this study aimed to provide insight into the understanding and experience of pearl millet among South African consumers and to determine the drivers and nudge points that could draw and attract consumers towards choosing pearl millet as an indigenous food source, turning an underutilised indigenous grain into a consumer-acknowledged food product of choice in support of a healthy lifestyle.

## 2. Materials and Methods

### 2.1. Research Design and Data Collection

**Figure 2:**
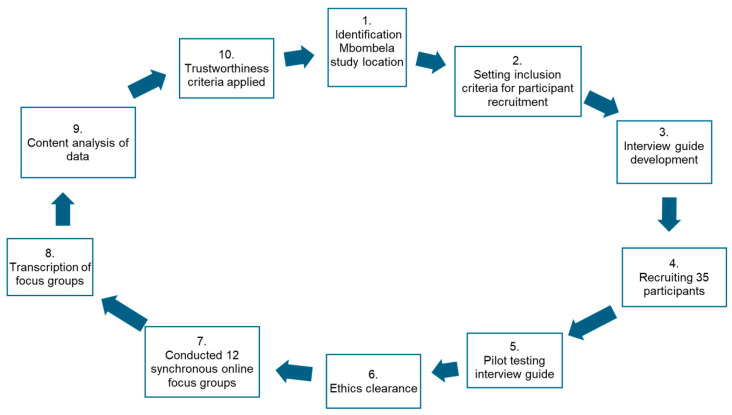
Qualitative research process followed to explore the understanding and experience of and attraction to pearl millet.

An interpretivist paradigm, allowing the participants to describe their experienced reality [58] within their societal context [59], in which a phenomenological exploratory study design was imbedded, was used to explore South African consumers’ understanding and subjective lived experience of pearl millet, as well as strategies to attract their attention to the grain. Following the research process (illustrated in Figure 2), the qualitative methodology used in this study allowed the researcher to gain knowledge about the pearl millet phenomena through small-group synchronous online focus-group discussions used to gather data. Keemink [60] points out that synchronous focus groups closely resemble in-person focus groups and are a valuable tool to examine participants’ thoughts and feelings about a topic [61]. By way of focus-group conversations, the researcher examined a small group of participants’ experiences, thoughts, and feelings about pearl millet in depth. The focus-group discussions were conducted in an online setting enabled by Microsoft Teams, which has been found to facilitate successful discussion, thus providing a promising method for online focus groups [60]. As this study was conducted during the COVID-19 pandemic, it was necessary to facilitate the focus-group discussions with minimum risk to the facilitator and participants, adhering to social distancing and the research protocol stipulated by the University of South Africa for conducting interviews.

Small focus groups specifically allow the participants to voice their opinions and express their emotions, thus facilitating more in-depth discussion [62]. Therefore, each focus group consisted of no more than three participants, resulting in 36 participants taking part in the study. The participants in this study resided mainly in Mbombela, which is situated in the province of Mpumalanga in South Africa. The participants, recruited through purposive, convenience, and snowball sampling methods, had to be older than 18 years, be the main persons responsible for purchasing food products in households, and have heard of pearl millet. Participants selected with these inclusion criteria would be more knowledgeable and aware of the presence of pearl millet in retail stores, which would enable them to talk about what they had come to understand about pearl millet. They also had to be customers of pharmacies, retail outlets, or health stores where pearl millet is sold, since that would mean a higher likelihood of them having been exposed to pearl millet. By setting these inclusion criteria for participation in the study, the researchers ensured that only participants who could discuss pearl millet based on their lived experience would be included. As a result, 12 synchronous online focus-group sessions, each approximately 45 to 60 min long, were held and recorded by way of Microsoft Teams. Most of the participants were between the ages of 18 and 39 years (94%), with Grade 12 as the highest education level. A high number of the participants (65%) were connected to the food industry in some way or other, with the remainder of the group not connected to the food industry at all. Data saturation was established after focus group 9 and confirmed in the additional focus groups that were conducted.

The co-investigator facilitated the focus groups. The interviews commenced after the piloting of the interview guide had taken place. The main questions that the participants responded to were as follows: (1) What do you know about indigenous grains and pearl millet? (2) What has been your experience of pearl millet as an indigenous grain? (3) What would you look for in a pearl millet product? The last question more specifically addressed attraction to pearl millet, which was considered through the perceived presence of external product attributes, such as price, packaging, country of origin, quality, and store image. This study received ethics clearance from the College of Agriculture and Environmental Sciences’ Health Research Ethics Committee on 27 January 2020, with the reference number 2020/CAES_HREC/016, prior to the commencement of the research. All the participants gave written consent before participating in the focus groups.

### 2.2. Data Analysis

All of the focus-group interviews were transcribed verbatim, after which each transcript was read several times. Thereafter, content analysis was performed to reduce the data. Open coding was first applied to identify segments of the data that captured the elements that were significant to the question asked. Through axial coding, categories that best represented the meaning of the codes were formed. The process was inductive in nature, whereby the researchers applied an emic stance to identify the opinions and expressions of the participants, as captured in the codes and categories. Verbatim quotes were used to support each category. Broad themes could then be assigned to the categories that were developed from the opinions of the participants. Johnson et al. [63] stress the importance of rigour and quality of data in qualitative research. That was achieved by utilising the trustworthiness criteria summarised by [64]. Credibility was achieved through prolonged engagement with the data and the participants in the study, peer debriefing during the code generation, and member checking during the focus-group interviews. The thick descriptive data obtained ensured transferability; dependability was addressed through a logical, traceable, and documented research methodology. That contributed to the confirmability of the data. Authenticity was achieved through audio recording and verbatim transcription of the interviews, with quotations specific to the responses of the participants used in the presentation of the data.

## 3. Findings and Discussion

The findings are discussed in relation to the participants’ understanding and experience of indigenous grains with reference to aspects that would attract consumers to choose to purchase pearl millet.

**Figure 3:**
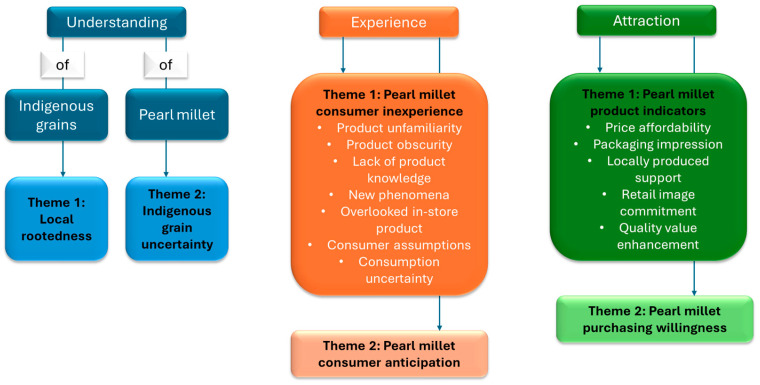
Thematic analysis of understanding and experience of and attraction to pearl millet.

The participants’ understanding of indigenous grains and pearl millet was examined by exploring the perceived meaning of the concepts of indigenous grains and pearl millet. From the data, two broad themes emerged: (i) the local rootedness of indigenous grains and (ii) uncertainty about indigenous grains. These are depicted in Figure 3 below.

### 3.1. Understanding Indigenous Grains and Pearl Millet

When exploring the concept of indigenous grains, the theme of local rootedness of indigenous grains emerged. It is possible that by attaching the meaning of a place-based human ethnic culture [65] to the word ‘indigenous’, the participants generally perceived indigenous grains as being ‘*from a specific area*’ and resembling ‘*grains which originated in our country, South Africa*’. Although ‘*not abundant*’, such grains were regarded as ‘*ancient grains*’, ‘*locally available*’, and ‘*locally grown*’. In addition, the immediate health-oriented perception of indigenous grains as ‘*specific types of grain that are healthy*’ emerged. Pearl millet was found [66] to be a rich source of resistant starch that supports a healthy colon [35] and is therefore advantageous to gastrointestinal health. It is not certain whether the participants based the latter perception on actual knowledge of indigenous grains regarded as nutrient-dense foods [9] and thus beneficial to the consumer.

Pearl millet is viewed as one of many crops widely grown and not considered a staple food [8], and ultimately not used to their full potential [4]. This can be ascribed to a lack of knowledge about the grain. When the participants considered the concept of pearl millet, the theme of uncertainty emerged, as the participants guessed ‘*it is a type of grain*’ or ‘*some sort…or kind of grain*’, thus only knowing that ‘*it is a grain found in South Africa*’ or thinking ‘*it’s a plant*’ or ‘*some form of a seed…or a grain or seed*’, perhaps ‘*something to do with food…some type of wheat, or similar to rice…or maize*’ or associating it with wheat that ‘*might be good for gut health*’ or perhaps a ‘*gluten-free grain*’, but ‘*nothing further than it being a grain*’. Any consumer doubt stemming from these responses could be addressed easily through informative communication and marketing campaigns, for which [54] continues to advocate. However, some of the participants were perceived to be more certain that it was ‘*underutilised*’, a ‘*type of grain*’, and ‘*used as staple food in underdeveloped countries*’, and that it is ‘*very filling*’ and could be used for ‘*livestock feed*’ or ‘*finely milled into flour and used in flour-based products*’.

The findings are further discussed with reference to the participants’ experience of the indigenous grain.

### 3.2. Experience of Pearl Millet

From the findings on the participants’ experience of pearl millet, two themes emerged, as depicted in Figure 3. Theme 1 is consumer inexperience of pearl millet stemming from perceived product unfamiliarity: not ‘*knowing much*’ or ‘*absolutely nothing*’ about the indigenous grain, and therefore ‘*I did not know that it exists*’. This unfamiliarity manifested in product obscurity, whereby participants ‘*have not seen these products in stores before*’, which could be attributed to statements like ‘*not something I grew up with*’ and, thus, no ‘*need for it*’. Consumer perceived familiarity with a food product establishes certainty about the product that results in an advantage over a novel, unfamiliar product. That could be the reason why consumers are perceived as not looking for pearl millet in stores [67]. Participant unfamiliarity with pearl millet resulted in a perceived lack of product knowledge and admission of not being ‘*aware of its benefits compared to normal grains or why it is superior*’. Participant inexperience with pearl millet also manifested in a lack of knowledge about its use: ‘*…many South African cookbooks, especially my grandma’s older cookbooks … never seen a recipe with it in*’, ‘*I don’t know how to incorporate them into my meals*’, and ‘*don’t know what do to with these products*’, leading to the non-purchase of the product: ‘*I only buy what I know*’. Through in-store cooking demonstrations, consumers could observe how new food products such as pearl millet could be incorporated into meals and consumer lifestyles [68]. Participant unfamiliarity with the grain further enhanced the perception that pearl millet was ‘*not a widely used product*’, ‘*not readily available everywhere*’, and, thus, ‘*not frequently used like other staple foods*’. However, De Groote et al. [69] did find an interest in fortified pearl millet products in low-income countries in Africa, and this finding is indicative of the role that pearl millet can play in food security. Among the participants, pearl millet was perceived to be a new phenomenon because of the higher interest in gluten-free products: ‘*all the gluten-free trends* [are] *increasing*’. The grain is thus more ‘*familiar around the new generation*’, ‘*known amongst farmers*’ and in ‘*rural communities…*[as] *a staple food*’, or only known to ‘*people in the food and beverage industry*’. Consequently, pearl millet has become an overlooked product in stores. Participants have ‘*not considered it when planning…weekly menus*’ and therefore not ‘*really looked for it*’. They also did not ‘*spend much time searching for new products*’ in-store, with a degree of consumer loyalty to familiar products found: ‘*I usually don’t stray from what I know*’ and ‘*only buy pastas and rice in the grain aisle in the stores*’. Furthermore, ‘*small town*[s] *only* [have] *basic groceries shops that don’t sell a wide variety of grains*’. Participants’ inexperience further manifested in perceived assumptions about pearl millet products, such as that they ‘*are usually more expensive than similar products such as wheat and maize*’ and ‘*quite expensive compared to samp or rice or pasta*’. Hence, it could be concluded that the perceived assumption that the participants shared was that pearl millet was an upmarket product not found in ordinary grocery stores in South Africa, but rather in ‘*health stores*’, ‘*wellness shops*’, ‘*pharmacies*’ where health foods are sold, or online: ‘*Health Connection sells it online*’. According to the study of [70], pearl millet can be processed into various food products, such as snacks, alcoholic and non-alcoholic beverages, boiled rice-like products, fermented and non-fermented bread, steam-cooked products, and thin or thick porridge. However, in considering the consumption of the grain by both animals and humans, producers have (in the case of humans) contemplated the aforementioned assumption and veered towards incorporating pearl millet as a component in specific food products that participants perceived to be ‘*porridge and cereal dishes*’; ‘*mueslis*’ or ‘*home-made trail mix*’; ‘*cereal bars*’; or ‘*as an ingredient in rice cakes…or puffed chips*’, ‘*gluten-free crackers and bread*’, ‘*vegan smoothies*’, or ‘*food bowls*’; hence, they have not considered it for consumption as a single grain source. Although very few of the participants perceived any other uses for pearl millet, it was mentioned that it was a suitable choice for health-related consumption in cases where ‘*people can’t consume gluten*’ or ‘*struggle with digestion and needs high-fibre intake*’. As a market growing faster than anticipated, the demand for gluten-free products has heightened regarding pearl millet as a potential gluten-free source [71]. Participants further assumed pearl millet to taste ‘*like raw oats*’, ‘*quinoa or couscous*’, ‘*nutty like seeds*’, ‘*bitter like some seeds…and bitter when eaten raw*’, and ‘*sandy and earthy*’, or to have ‘*quite a neutral taste*’ that is ‘*like most seeds*’. In this regard, it was found that substituting wheat flour with pearl millet flour in peanut cookies did not result in a significant difference in organoleptic quality, suggesting that the taste of products with pearl millet may not result in the negative taste participants expected [72]. A further result of inexperience with pearl millet is consumption uncertainty, as participants were ‘*not sure if I have*’ or thought that they had ‘*most probably*’ consumed pearl millet without knowing for certain.

Even though the participants perceived pearl millet as a new product not well known among South African consumers, their uncertainty about this indigenous grain did not seem to close them off to being introduced to pearl millet. That resulted in the emergence of the second theme under experience, as reflected in Figure 3: pearl millet product anticipation. In this regard, some participants were rather keen and ‘*pretty open to try new things*’ and ‘*new food products*’ such as pearl millet, but pointed out that it would depend on whether ‘*the quality looks good*’. Consumers who are open to new food products have higher purchase intent and acceptance of novel foods [73], which could include pearl millet products as a choice product. This suggests that an indigenous grain like pearl millet should be presented as a superior product and not as a cheap, unattractive grain. However, not all the participants were keen to try new products, since they ‘*hate trying new things*’ because they did not ‘*really splurge on new things that are a risk of being not pleasant to eat*’. In this instance, it was suggested that a ‘*smaller packet size for tasting purposes would be great when trying a new product, instead of buying a big bag of something that you are not sure if you would like*’. It could therefore be concluded that it is essential that the quality of pearl millet and its presentation to the consumer are convincing and less risky in order to make it a product of choice.

Finally, the findings are discussed with reference to aspects that would attract consumers to pearl millet.

### 3.3. Attraction to Pearl Millet

The findings on the attributes that would attract consumers to purchase pearl millet resulted in two general themes emerging that identified specific pearl millet product indicators and pearl millet purchase-willingness indicators as the underlying drivers of pearl millet attraction (Figure 3). In relation to the theme of pearl millet product indicators, price affordability would serve to attract consumers to pearl millet. In addition to affordability, the emphasis should be on pearl millet being a product that ‘*gives me value for money…not overpriced*’. As a result, participants expected the ‘*price to be similar to alternative products*’ or ‘*other cereal grains*’: ‘*if it seems…it would give me that, I will consider buying the product*’. Al-Salamin and Al-Hassan [74] found that the more suitably priced a product is, the more willing consumers are to purchase it. Participants cautioned that the ‘*price should not be outrageous*’ but ‘*affordable and give me value for money*’; the product is expected to be affordable, ‘*as it is an alternative to staple foods in South Africa*’.

The second attraction indicator was packaging impression. Informative and educational packaging ‘*telling me more about pearl millet*’ that provided ‘*cooking uses and recipe ideas*’, ‘*instruction for use*’, ‘*what it is good for*’, ‘*instructions on storage*’, and ‘*nutritional information*’ would attract the attention of consumers. Such information should preferably be presented in a ‘*descriptive and also visual*’ manner, with ‘*pictures*’. In general, the participants commented on a sensation of enjoyment results when ‘*packaging catches my eye by being neat, modern and different*’. Thus, ‘*something simple yet elegant to separate pearl millet from other products*’ was advised. Packaging, as an acknowledged mechanism to attract consumer attention [75], would therefore be vital to the introduction of an indigenous grain such as pearl millet. Product visibility was an even more important consideration for product packaging. Participants explained: ‘*I would like to see the product in* [the] *packaging and not just a picture*’ when pearl millet product packaging is being developed. Another general packaging requirement that the participants would apply to measure food products was the functionality of the packaging, including aspects like ‘*firm packaging protecting the product*’ supported by ‘*sturdy wrapping, not necessarily carton boxed*’, with ‘*as little plastic, as possible*’, where the ‘*packaging…aids the environment*’ in addition to ‘*packaging reseal convenience*’. Informative packaging could assist the consumer in becoming more familiar with the product and its uses.

The third component under attraction was that pearl millet as an indigenous grain seemed to evoke locally produced support through ‘*keen*[ness] *to support locally produced products*’. To some of the participants, it was ‘*important*’ that such products ‘*must be proudly South African*’ because they were ‘*inclined to buy locally produced products*’. Although some participants mentioned that it was not always ‘*the first thing I would notice on a label*’, for others, the inclusion of the country of origin of a product such as pearl millet on the packaging ‘*will give me a good idea if I want to buy it or not*’. This inclination has been confirmed by [76] as a factor considered by consumers when they do not know much about a product. The participants also indicated that they were in favour of a locally produced indigenous grain because of their willingness to support the local economy. Participants stated that ‘*local is better and helps the economy more*’ and that ‘*anything affecting the economy of my country is important and I would support it*’. This support was further relayed to the farmer as the producer of pearl millet who would benefit from participants supporting local produce: ‘[I] *like to purchase South African products to support the farmers in our country*’. Although product labelling reflecting country of origin was relevant for some participants, others might ignore it because ‘*I don’t really look at the label*’. And for some, it could have little influence on their purchase decision, as it seemed to be ‘*the last thing* [they] *would look at*’, and thus it ‘*does not really affect me*’. In this regard, Yang [77] believes that in an increasingly borderless world, the country of origin is an insignificant factor in the product purchases of consumers. However, its pronouncement as a proudly South African product could be a positive factor in encouraging consumer interest in the product.

Retailer image commitment was a further aspect that would ensure attraction to pearl millet. Consumers could expect a retailer’s in-store brand experience to support the promotion of pearl millet. A positive experience would distinguish a retailer from its competitors [78]. Participants stated the following in this regard: ‘*If the store image has a good reputation…I will be inclined to try* [a] *new product*’ because a ‘*retail image sells the product*’ and ‘*inspires confidence in the product being sold*’ through a ‘*commitment to selling quality products*’. Participants also indicated that if a store provided store ambience, whereby ‘*the look and feel is great, I would be more likely to explore the store*’, resulting in ‘*…more time I would spend browsing in-store*’ and, thus, greater willingness to ‘*try new products*’ such as pearl millet. Successful consumer introduction to an indigenous product could therefore rely on retailers’ ability to promote and associate with a product such as pearl millet in a way with which consumers can relate. Participants pointed out that ‘*promotions for new products*’ and ‘*in-store tastings are a good way to connect to customers and present new products*’. Demonstrating ‘*different ways that it can be made*’ is especially important because ‘*cool new recipes that have the unfamiliar product in*’ would enhance attraction to pearl millet as an indigenous grain.

Product attraction would further be enhanced through the quality-value enhancement of pearl millet, which was the ‘*main and only factor of influence*’ to some of the participants who ‘*rely on quality as key factor when I don’t know anything of the product*’ and ‘*if quality is bad, I won’t repurchase it*’. Of greater importance is the finding that price somehow reflects the quality of the product: ‘*If* [the] *price is low, I would think that quality is cheap*’, thus confirming the notion that price is an expression of product value [79]. Although attaching quality to price, participants still required pearl millet to be ‘*value for money*’, as reflected by the following statement: ‘*I would buy the product if it gives me good value for money, I expect the quality to be good and judge price therefore*’. Quality was also deduced from the visual appeal of the product: ‘*The look and feel represent quality*’, as ‘*I eat with my eyes, so if it looks unappealing, I won’t buy it*’ because ‘*if it looks cheap it will taste cheap*’. The findings therefore indicate that the quality of pearl millet is not based on its internal composition as such, but rather on appearance and cost.

Concerning the theme of willingness to purchase pearl millet, a further indicator suggested the need for pearl millet advocacy to attract the attention of South African consumers. Participants pointed out that greater awareness of the product should be created through ‘*good branding*’ and ‘*good advertising*’ that would establish ‘*awareness of the product outside of the healthy food spectrum*’. That would assist in consumers noticing pearl millet. In South Africa, pearl millet is predominantly found in health stores and less in general food retail stores. However, irrespective of where pearl millet is sold, participants revealed that the healthfulness of pearl millet was another consideration for product purchase: ‘*I will consider it if it has the health benefits I am looking for*’, meaning that it is a ‘*good nutrient-dense food*’ and the ‘*nutritional value is good for my family*’. The advocacy of pearl millet may be enhanced by favourable product reviews and peer expertise, and trustworthiness has been found to be a socially significant influencer of consumer purchasing behaviour that could influence pearl millet consumption significantly [80]. As such, reviews on social media or recommendations by ‘[a] *friend or family*’ would influence purchase willingness, with participants indicating that they would consider purchasing pearl millet ‘*if the reviews are great*’. As mentioned previously, product affordability remained a critical element in the decision to purchase pearl millet. An ‘*affordable price*’ would assist the participants in determining whether pearl millet was ‘*value for money*’, an aspect that was of special importance when ‘*I am not familiar with the product and I don’t want to be wasting my money*’. The product should also be ‘*affordable to all South Africans*’. Such cautious behaviour may relate to the level of perceived financial risk associated with a newly introduced product [81], such as pearl millet. However, in contrast, participants highlighted the importance of price sensitivity in respect to quality, with them indicating that ‘*if the price is low, I would think the quality is cheap*’. Affordability was supported by the requirement of product availability. ‘*The product should be readily available*’, since that would enhance the willingness to purchase it. However, product quality remained a key element in influencing willingness to purchase pearl millet because ‘*if quality is bad, I won’t purchase it* [again]’. The successful introduction of pearl millet would therefore rely heavily on the quality of the product because some consumers may ‘*rely on quality as* [the] *key factor when I don’t know anything of the product*’. For such consumers, quality would be ‘*the main and only factor of influence*’. Apart from playing an important role in attraction to the product, its visual appearance would contribute to the willingness to purchase. When introduced to pearl millet, ‘*I would look at visual cleanliness of the product*’ because ‘*I eat with my eyes. So, if it looks unappealing, I won’t buy it*’ and ‘*if it looks cheap, it will taste cheap*’. As already mentioned, Wang [79] found that price was regarded as an expression of product value that influenced willingness to purchase a product significantly [82]. In that sense, ‘*price goes hand in hand with the quality of the product*’, thus representing an important element that would also influence the willingness to purchase pearl millet. Additionally, the ‘*presentation of the product is crucial*’ because ‘*the look and feel represent quality*’. Willingness to purchase pearl millet would also be determined by product information that consumers use as ‘*background information on how it is grown, treated, preserved, et cetera*’. ‘*Packaging information on storage use*’, as well as visible ‘*certification logos*’ and ‘*descriptive information*’, would therefore assist the consumer in arriving at a decision about purchasing pearl millet products. Consumer knowledge seemed to influence familiarity and, thus, willingness to purchase pearl millet because ‘*the more knowledge I have, the more I would consider it*’. That included providing ‘*interesting facts on the packaging*’ and ‘*recipe guidance*’.

## 4. Conclusions

This study aimed to provide insight into South African consumers’ understanding and experience of pearl millet and to determine the drivers and nudge points that could direct and attract consumers to choose pearl millet as an indigenous food source.

1. The implementation of pearl millet drivers and nudge points could change the underutilisation of this indigenous grain into a consumer-acknowledged food product of choice that supports a healthy lifestyle for both food-secure and food-insecure consumers.

The elevation of South Africa’s pearl millet grain from being produced by subsistence farmers for indigenous use to a food product found in health stores represents vast extremes. The perplexing attainment value being attributed to pearl millet has placed it out of reach of ordinary South African consumers, while it should be a grain that they purchase regularly to add diversity to their diets. The findings of this study suggest that consumers’ unfamiliarity with pearl millet may influence their experience of this grain strongly. An indigenous grain that has been around for decades has become unfamiliar to the modern consumer. That has resulted in consumer inexperience, with pearl millet being overlooked in stores and not purchased for the nutritional benefits it has.

2. To address consumer unfamiliarity with pearl millet, marketing has to create more awareness of and a better image for pearl millet, which would result in consumer confidence in choosing the grain.

The hidden inclusion of pearl millet in upmarket health bars or other health-related food products polarises the market on a much-needed grain that could change the dietary intake of many South African consumers.

3. Consumers must be educated and exposed to products containing pearl millet and provided with recipes in which it can be used if consumer consumption and choice are to be improved.

Consumer anticipation of pearl millet presented as a quality product, in manageable proportions to test out, was evident in the keenness to gain more experience about pearl millet. This implies that the unfamiliarity of pearl millet may result in cautious consumers who are afraid of investing in pearl millet only to be disappointed. Unfortunately, pearl millet was never meant to be an exclusive grain. Historically, it provided sustenance to subsistence farmers and added nutritional value to the meals of those who consumed it.

4. Specific product indicators would tempt consumers to purchase pearl millet, but without the right attractions, such as price, packaging, an emphasis on locally produced, retailer image, and quality perceptions, all attempts to increase consumption may continue to be in vain.

Pearl millet should be marketed for what it is, which is a powerful indigenous grain that should cost the consumer less in terms of environmental and financial resources because it is locally produced and should be supported for that purpose. There is a willingness among consumers to choose pearl millet as an indigenous grain, but that is subject to manufacturers and marketers addressing the indicators identified by the participants as prerequisites for them to consider pearl millet as a grain to purchase.

5. Without the required product indicators consumers specified, pearl millet will remain unnoticed and undervalued.

This study contributes to a better understanding of South African consumers’ position in terms of pearl millet and what is needed to get them interested in and attracted to choose this grain—aspects that have not been studied before. The study was limited to South African views on pearl millet. Future research may need to expand to include a broader African-continent consumer-choice perspective and quantify the attraction indicators to determine the importance of these indicators for many other consumers in Africa. Research may also need to determine the importance of pearl millet to the urban consumer in order to fully understand the reason why urban consumers may not be attracted to this grain. That could provide marketers with the nudging elements to improve pearl millet choice and consumption within this market. South Africa and Africa can ill afford to allow an indigenous grain such as pearl millet, which has the potential to address SDG2 and SDG3, to remain underutilised. Current economic conditions and financial constraints facing consumers in South Africa also warrant that attention be focused on an indigenous grain such as pearl millet, because it is a grain that could feed the nation if the misconceptions about, stigmatisation of, and consumer uncertainty and inexperience of the grain could be addressed.

## Data Availability

The data is available from the corresponding author after consideration of the request.
